# An Efficient Approach to the Synthesis of Highly Congested 9,10-Dihydrophenanthrene-2,4-dicarbonitriles and Their Biological Evaluation as Antimicrobial Agents

**DOI:** 10.3390/molecules181215704

**Published:** 2013-12-16

**Authors:** Hassan M. Faidallah, Khalid M. A. Al-Shaikh, Tariq R. Sobahi, Khalid A. Khan, Abdullah M. Asiri

**Affiliations:** 1 Department of Chemistry, Faculty of Science, King Abdulaziz University, P.O. Box 80203, Jeddah 21589, Saudi Arabia; E-Mails: khalidyat@hotmail.com (K.M.A.A.-S.); drtariq_s@hotmail.com (T.R.S.); kzsakhan@hotmail.com (K.A.K.); aasiri2@gmail.com (A.M.A.); 2 Center of Excellence for Advanced Materials Research, King Abdulaziz University, P.O. Box 80203, Jeddah 21589, Saudi Arabia

**Keywords:** dihydrophenanthrene, X-ray crystallography, dihydrobenzo[h]quinoline-3-carbonitrile, antimicrobial activity

## Abstract

An efficient and novel method for the synthesis in moderate to good yield (72%–84%) of a series of 3-amino-1-substituted-9,10-dihydrophenanthrene-2,4-dicarbonitriles **1**–**5** via one-pot multi-component reactions of aldehydes, malononitrile, 1-tetralone and ammonium acetate has been delineated. Cyclocondensation attempts of aminocyanophenanthrene derivatives **1**, **2**, **4** and **5** with acetic anhydride in the presence of conc. H_2_SO_4_ failed and instead the diacetylamino derivatives **10**–**13** were obtained. All prepared compounds were structurally elucidated by various spectroscopic methods and X-ray crystallography. *N*,*N*-diacetylamino-derivatives of phenanthrene have shown good antimicrobial activity.

## 1. Introduction

The phenanthrene moiety is a structural component of many natural products and has been linked to important roles in the pharmaceutical and biological realms. It exhibits a broad spectrum of biological activities such as antimalarial [1], anticancer [2] antimycotic [3,4] anti-HIV [5] and emetic properties [6]. C9-substituted phenanthrene-based tylophorine derivatives have shown potential antiviral activity against tobacco mosaic virus (TMV) [7]. In addition, 3,7-dihydroxy-2,4,8-trimethoxyphenanthrene is known for its anti-inflammatory properties [8], while the derivative aristolochic acid exhibits tumor-inhibitory potential [9]. The phenanthrene derivatives 3-hydroxy-2,4-dimethoxy-7,8-methylenedioxyphenanthrene and 2,7-dihydroxy-1-methyl-5-vinylphenanthrene isolated from the rhizomes of *Tamus communis* exhibit cytotoxicity [10,11]. 9,10-Dihydrophenantherenes with pendant carboxyl groups at position-2 are reported as inhibitors of 5α-reductase and useful in the treatment of pharmacological disorders associated with elevated levels of dihydrotestosterone [12].

Various methods have been reported for the synthesis of phenanthrenes [13] and dihydrophenanthrenes [14,15,16,17,18,19,20] through ring annulation [21], intermolecular [22] and intramolecular [23] cyclization. A majority of the procedures have certain limitations of accessibility of the precursors, require multiple steps, have harsh reaction conditions, are incompatible with the presence of functional groups, have relatively overall low yields, and lack well-defined regiocontrol elements. Phenanthrenes were prepared from disubstituted biphenyls by intramolecular condensation [22], metal-catalyzed rearrangement of alkene-alkynes [24] and cycloisomerization [25] depending on the functionality present in the biphenyl moiety. Palladium-catalyzed cyclization of arynes with alkynes is an alternative route for the synthesis of phenanthrene [24] and 9,10-disubstituted phenanthrene derivatives [26].

The diverse pharmacological activities and limitations of convenient and efficient procedures prompted us to develop a concise, straight forward and economical route to the synthesis of this class of compounds without use of any catalyst.

## 2. Results and Discussion

### 2.1. Chemistry

**Scheme 1:**
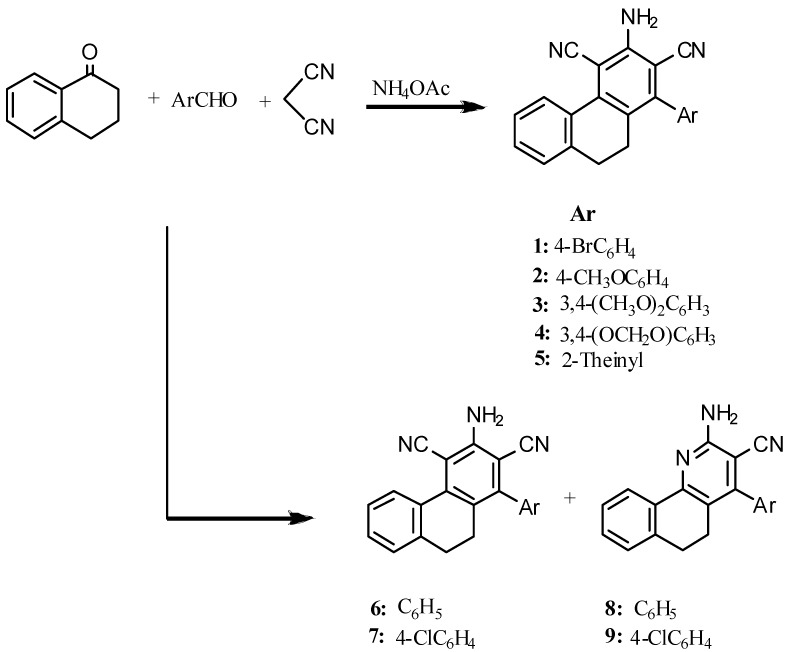
Synthesis of Compounds **1**–**9**.

**Scheme 2:**
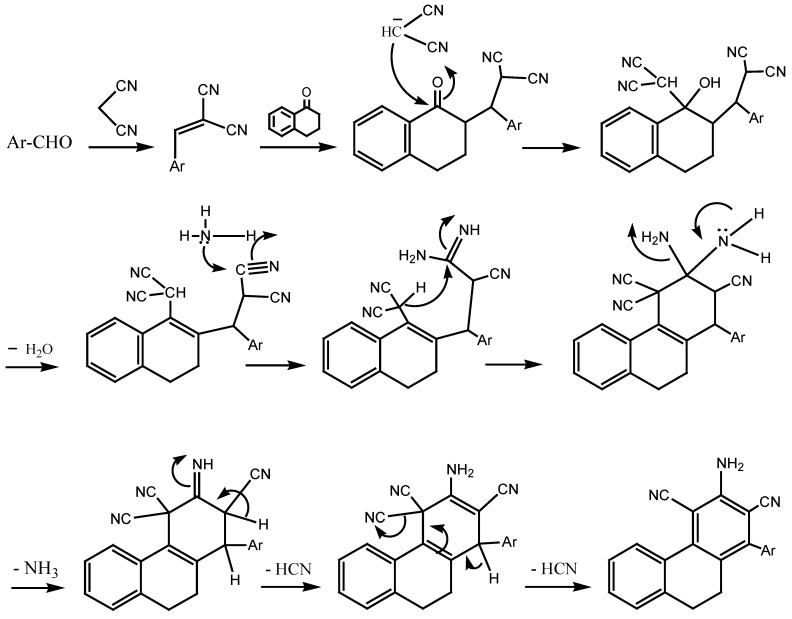
A possible mechanism of the formation of 9,10-dihydrophenanthrene-2,4-dicarbonitriles.

**Figure 1:**
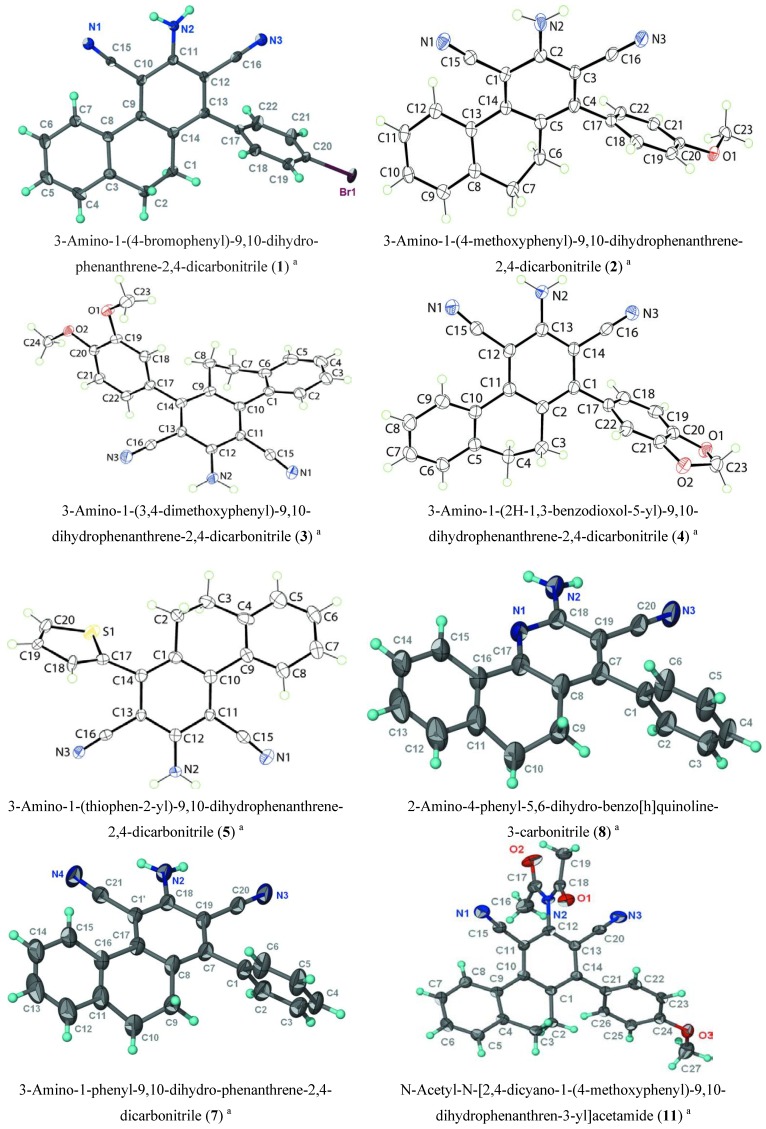
X-ray crystal structures of some dihydrophenanthrenes and dihydrobenzoquinolines.

^a^ Source: reprinted from [29,30,31,32,33,34,35,36].

It has been reported [27,28] that 5,6-dihydrobenzo[h]quinoline derivatives are prepared by condensation of the corresponding 2-arylidene-1-tetralone with malononitrile in the presence of ammonium acetate or via one-pot multicomponent reactions (MCRs) of aldehydes, malononitrile, 1-tetralone and ammonium acetate. However, we found that one-pot MCRs of aldehydes, malononitrile, 1-tetralone and ammonium acetate yielded the corresponding phenanthrene derivatives instead of the expected 5,6-dihydrobenzo[h]quinolines (Scheme 1). The probable mechanism for the formation of phenanthrene derivatives is shown in Scheme 2. The structures of these phenanthrene derivatives were elucidated by analytical (Table 1) and spectroscopic data (see Experimental). The conclusive proof of the phenanthrene structure over the quinoline one was given by X-ray crystallography (Figure 1).

**Table 1:** Physical and analytical data of compounds **1**–**13**.

Compd.	R	Yield	m.p.	Mol. Formula	Calc. %			Found %		
		(%)	(°C)		C	H	N	C	H	N
**1**	4-BrC₆H₄	76	244–246	C_22_H_14_BrN_3_	66.01	3.53	10.50	65.98	3.64	10.45
**2**	4-CH_3_OC₆H₄	82	214–216	C_23_H_17_N_3_O	78.61	4.88	11.96	78.48	4.68	11.89
**3**	3,4-(CH_3_O)_2_C₆H_3_	78	266–268	C_24_H_19_N_3_O_2_	75.57	5.02	11.02	75.62	5.16	10.87
**4**	3,4-(OCH_2_O) C₆H_3_	84	276–278	C_23_H_15_N_3_O_2_	75.60	4.14	11.50	75.52	4.20	11.42
**5**	2-Thienyl	72	220–222	C_20_H_13_N_3_S	73.37	4.00	12.83	73.42	3.89	12.72
**6**	C_6_H_5_	32	340–343	C_22_H_15_ N_3_	82.22	4.70	13.08	82.34	4.65	13.12
**7**	4-ClC₆H₄	49	238–240	C_22_H_14_Cl N_3_	74.26	3.97	11.81	74.41	4.02	11.68
**8**	C_6_H_5_	50	290–293	C_20_H_15_ N_3_	80.78	5.08	14.13	80.82	5.11	14.23
**9**	4-ClC₆H₄	12	222–225	C_20_H_14_Cl N_3_	72.40	4.25	12.66	72.36	4.32	12.61
**10**	4-BrC₆H₄	82	275–277	C_22_H_14_BrN_3_	66.01	3.53	10.50	66.12	3.51	10.75
**11**	4-CH_3_OC₆H₄	80	344–346	C_27_H_21_N_3_O_3_	74.47	4.86	9.65	74.61	4.68	9.55
**12**	3,4-(OCH_2_O) C₆H_3_	77	265–267	C_27_H_19_N_3_O_4_	72.15	4.26	9.35	72.23	4.08	9.19
**13**	2-Thienyl	76	355–357	C_24_H_17_N_3_O_2_S	70.05	4.16	10.21	70.12	4.22	10.31

It is worthy to mention here that when the aromatic aldehyde is benzaldehyde, or 4-chlorobenzaldehyde two products were isolated; one is the expected benzoquinoline derivative **8** or **9** and the other is the unexpected phenanthrene **6** or **7**. However, in case of the 4-chlorobenzaldehyde the major product is the phenanthrene derivative (phenanthrene/benzoquinoline 4:1) while with benzaldehyde the benzoquinoline derivative is the major product (benzoquinoline/phenanthrene 5:3).

**Scheme 3:**
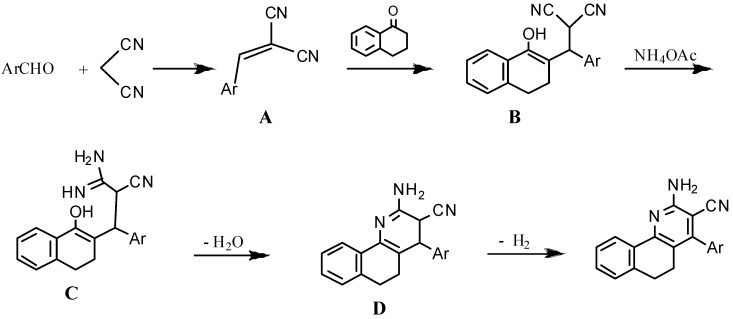
A possible mechanism of the formation of benzoquinoline derivatives.

The formation of the benzoquinoline may be explained according to the following mechanism (Scheme 3). The reaction seemed to be started by first addition of active hydrogen of 1-tetralone to the ethylenic double bond of compound **A**. Ammonia was added to the nitrile group in **B** to give **C** which looses a molecule of water to give **D**, which in turn was converted to the final product by auto-oxidation.

The IR spectra of compounds **1**–**9** revealed absorption bands at 3,359–3,389 cm^−1^ characteristic for the NH_2_ and at 2,318–2,226 cm^−1^ attributed to the CN group. Their ^1^H-NMR spectra exhibited, besides the aromatic protons at δ 7.17–8.02, two multiplets at δ 2.86–2.94 and 2.80–2.85 ppm corresponding to the benzylic protons (C^9^-*H* and C^10^-*H* respectively) as well as an exchangeable NH_2_ at δ 6.54–6.67. The structures were further supported by ^13^C-NMR which showed in addition to the expected number of aromatic carbons, two signals at δ 34.2–34.7 and 26.2–26.8 for the benzyl carbons (C-9 and C-10 respectively). More evidence for the structures of compounds **1**–**9** arise from their X-ray crystallography [29,30,31,32,33,34,35] data which confirm the phenanthrene structure for compounds **1**–**5** (Figure 1). However, when Ar = Ph or 4-Cl-C_6_H_4_, the X-ray crystallography confirms that they are a mixture of the benzoquinoline derivatives **6** and **7** and corresponding phenanthrene **8** and **9**.

**Scheme 4:**
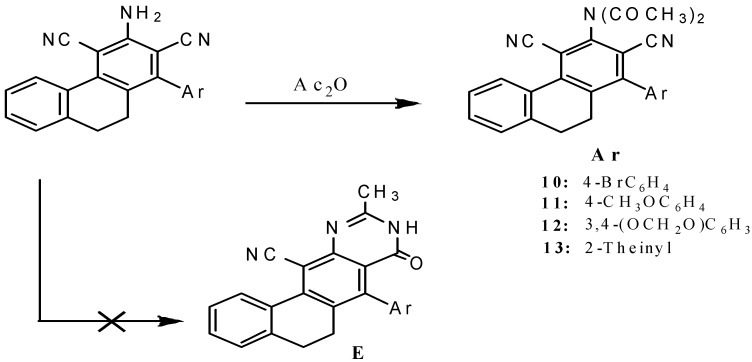
*N*,*N*-Diacetyl derivatives of dihyrophenenthrene **10**–**13**.

Attempts were made to prepare 2-methylpyrimidone derivative **E** by cyclization of the 3-aminophenanthrene derivatives **1**, **2**, **4** and **5** with acetic anhydride either in the presence or the absence of conc. H_2_SO_4_ adopting the same procedure reported in the literature [37,38,39,40], but these reactions afforded the *N*,*N*-diacetylaminophenanthrenes **10**–**13** instead of the expected pyrimidone derivatives (Scheme 4).

The IR spectra of compounds **10**–**13** were characterized by the absence of the NH_2_ absorption and the presence of two new sharp absorption bands in the 1,666–1,672 cm^−1^ region due to the new C=O groups. Their ^1^H NMR exhibited, besides aromatic protons at δ 7.14–7.48, two multiplets at δ 2.82–2.86 and 2.84–2.88 ppm corresponding to the H-9 and H-10 respectively, as well as a singlet of six proton intensity at δ 2.40–2.44 for the 2CH_3_ groups. The structures were further supported by ^13^C-NMR data which showed in addition to the expected number of aromatic carbons, two signals at δ 34.0–34.5 and 26.2–26.6 for C-9 and C-10 in addition to the CH_3_ carbons at δ 14.7–14.9. More evidence for the structure of compound **11** comes from its X-ray crystallography [36].

### 2.2. Biological Evaluation

#### 2.2.1. *In Vitro* Antibacterial and Antifungal Activities

Compounds **1**–**13** were screened *in vitro* for their antimicrobial and antifungal activities against *Escherichia coli*, *Staphylococcus aureus*, *Aspergillus niger* and *Candida albicans*. The zones of inhibition formed for the compounds against bacteria and fungi are summarized in Table 2. The overall results suggested that compounds containing 3-(*N*,*N*-diacetylamino)-substituent exhibited relatively better antimicrobial and antifungal activities when compared with non-acetylated phenanthrene analogs thereby indicating a positive role of diacetyl-substitution in the present series. Compounds **10** and **13** were however, significantly active when compared with rest of the series. All test data in Table 2 were of average value from triplicate runs and the test compounds showed reduced antimicrobial activities when compared with their respective standards.

**Table 2:** Anti-bacterial and anti-fungal data of compounds **1**–**13**.

Compound	Zone of Inhibition in mm		Zone of Inhibition in mm	
	*Escherichia coli*	*Staphylococcus aureus*	*Aspergillus niger*	*Candida albicans*
**1**	25	24	18	21
**2**	21	20	15	18
**3**	20	21	14	16
**4**	23	20	13	18
**5**	25	22	16	20
**6**	16	15	12	13
**7**	18	17	14	15
**8**	12	13	10	13
**9**	14	15	12	15
**10**	30	28	20	22
**11**	28	26	17	19
**12**	26	25	19	20
**13**	29	29	21	22
**Ampicillin**	30	29	-	-
**Clotrimazole**	-	-	33	32

## 3. Experimental

### 3.1. General Methods

Melting points were determined on a Gallenkamp melting point apparatus and are uncorrected. The infrared (IR) spectra were recorded on Shimadzu FT-IR 8400S infrared spectrophotometer using the KBr pellet technique. ^1^H- and ^13^C-NMR spectra were recorded on a Bruker DPX-400 FT NMR spectrometer using tetramethylsilane as the internal standard and DMSO-*d_6_* as a solvent (chemical shifts in δ, ppm). Splitting patterns were designated as follows: *s*: singlet; *d*: doublet; *m*: multiplet; *q*: quartet. Elemental analyses were performed on a 2400 Perkin Elmer Series 2 analyzer and the found values were within ±0.4% of the theoretical values. Follow up of the reactions and checking the homogeneity of the compounds were made by TLC on silica gel pre-coated aluminum sheets (Type 60 F254, Merck) and the spots were detected by exposure to UV-lamp at λ 254. 

#### 3.1.1. 3-Amino-1-substituted-9,10-dihydrophenanthrene-2,4-dicarbonitriles **1**–**5**

A one-pot mixture of the appropriate aldehyde (10 mmol), 3,4-dihydro-2*H*-naphthalene-1-one (1.46 g, 10 mmol), malononitrile (0.66 g, 10 mmol) and ammonium acetate (6.2 g, 80 mmol) in absolute ethanol (50 mL) was refluxed for 6 h. The reaction mixture was allowed to cool, and the resulting precipitate was filtered, washed with water, dried and recrystallized from DMF. Yields, 72%–84%; IR: (cm^−1^, KBr): 3357–3361, 3420–3428 (NH_2_), 2220–2231 (CN). 

*3-Amino-1-(4-bromophenyl)-9,10-dihydrophenanthrene-2,4-dicarbonitrile* (**1**): ^1^H-NMR (DMSO-*d*_6_) δ ppm: 2.86 (m, 2H, C-9 protons), 2.80 (m, 2H, C-10 protons), 7.12 (s, 1H, NH_2_), 7.24–7.48 (m, 9H, Ar-H). ^13^C-NMR (DMSO-*d*_6_) δ ppm: 34.2 (C-9), 26.3 (C-10), 116.6 (CN), 97.5, 126.2, 126.4, 127.3, 127.5, 128.6, 129.1, 129.5, 136.2, 136.7, 139.0, 145.2, 152.0 (Ar-C).

*3-Amino-1-(4-methoxyphenyl)-9,10-dihydrophenanthrene-2,4-dicarbonitrile* (**2**): ^1^H-NMR (DMSO-*d*_6_) δ ppm: 2.88 (m, 2H, C-9 protons), 2.83 (m, 2H, C-10 protons), 3.74 (CH_3_O), 6.98 (s, 1H, NH_2_), 6.85–7.49 (m, 8H, ArH). ^13^C-NMR (DMSO-*d*_6_) δ ppm: 33.8(C-9), 26.2 (C-10), 56.1 (CH_3_O), 116.5 (CN), 97.4, 114.4, 126.2, 126.6, 127.5, 128.4, 128.7, 129.4, 136.1, 139.4, 145.2, 151.8, 160.6 (Ar-C). 

*3-Amino-1-(3,4-dimethoxyphenyl)-9,10-dihydrophenanthrene-2,4-dicarbonitrile* (**3**): ^1^H-NMR (DMSO-*d*_6_) δ ppm: 2.86 (m, 2H, C-9 protons), 2.82 (m, 2H, C-10 protons), 3.72 (CH_3_O), 3.73 (CH_3_O), 6.87 (s, ^1^H, NH_2_), 6.72–7.50 (m, 7H, Ar-H). ^13^C-NMR (DMSO-*d*_6_) δ ppm: 34.1 (C-9), 26.4 (C-10), 56.2 (CH_3_O), 56.3 (CH_3_O), 117.0 (CN), 97.3, 114.0, 115.5, 116.3, 120.7, 126.3, 127.4, 128.4, 129.6, 129.7, 135.9, 139.1, 145.2, 146.4, 148.2, 151.8 (Ar-C). 

*3-Amino-1-(benzo[d][1,3]dioxol-5-yl)-9,10-dihydrophenanthrene-2,4-dicarbonitrile* (**4**): ^1^H-NMR (DMSO-*d*_6_) δ ppm: 2.87 (m, 2H, C-9 protons), 2.80 (m, 2H, C-10 protons), 5.91(CH_2_), 6.86 (s, 1H, NH_2_), 6.80–7.48 (m, 7H, Ar-H). ^13^C-NMR (DMSO-*d*_6_) δ ppm: 34.2 (C-9), 26.5 (C-10), 117.2 (CN), 91.2 (CH_2_), 97.5, 114.2, 115.6, 116.4, 120.7, 126.2, 127.6, 128.5, 129.4, 129.9, 136.0, 139.1, 145.3, 146.2, 148.4, 151.9 (Ar-C).

*3-Amino-1-(thiophen-2-yl)-9,10-dihydrophenanthrene-2,4-dicarbonitrile* (**5**): ^1^H-NMR (DMSO-*d*_6_) δ ppm: 2.89 (m, 2H, C-9 protons), 2.81 (m, 2H, C-10 protons), 6.75 (s, 1H, NH_2_), 6.98–7.47 (m, 7H, Ar-H). ^13^C-NMR (DMSO-*d*_6_) δ ppm: 34.3 (C-9), 26.3 (C-10), 116.9 (CN), 97.2, 98.6, 122.8, 125.2, 126.3, 127.4, 127.5, 128.3, 129.1, 136.0, 139.1, 142.4, 145.2, 151.8 (Ar-C).

#### 3.1.2. 3-Amino-1-substituted-9,10-dihydrophenanthrene-2,4-dicarbonitriles **6** and **7** and 2-Amino-4- substituted-5,6-dihydrobenzo[h]quinoline-3-carbonitriles **8** and **9**

A mixture of the 1-tetralone (1.46g, 10 mmol), the appropriate aldehyde (10 mmol), malononitrile (0.66 g, 10 mmol) and ammonium acetate (6.2 g, 80 mmol) in absolute ethanol (50 mL) was refluxed for 3–6 h. The reaction mixture was cooled and the resulting precipitate was filtered, washed with water, dried. The obtained solid was then subjected to column chromatography on silica gel (n-hexane/ethyl acetate 9:1) to give two products. IR: (cm^−1^, KBr): 3367–3372, 3429–3435 (NH_2_), 2219–2224 (CN).

*3-Amino-1-phenyl-9,10-dihydrophenanthrene-2,4-dicarbonitrile* (**6**): ^1^H-NMR (DMSO-*d*_6_) δ ppm: 2.86 (m, 2H, C-9 protons), 2.80 (m, 2H, C-10 protons), 7.12 (s, 1H, NH_2_), 7.24–7.48 (m, 9H, Ar-H). ^13^C-NMR (DMSO-*d*_6_) δ ppm: 34.2 (C-9), 26.2 (C-10), 116.6 (CN), 97.5, 126.2, 126.4, 127.3, 127.5, 128.6, 129.1, 129.5, 136.2, 136.7, 139.4, 145.2, 152.0 (Ar-C).

*3-Amino-1-(4-chlorophenyl)-9,10-dihydrophenanthrene-2,4-dicarbonitrile* (**7**): ^1^H-NMR (DMSO-*d*_6_) δ ppm: 2.84 (m, 2H, C-9 protons), 2.81 (m, 2H, C-10 protons), 7.03 (s, 1H, NH_2_), 7.16–7.47 (m, 9H, ArH). ^13^C-NMR (DMSO-*d*_6_) δ ppm: 33.3 (C-9), 26.9 (C-10), 117.4 (CN), 97.5, 126.1, 126.4, 127.0, 127.5, 128.4, 129.3, 129.6, 136.2, 137.2, 139.6, 145.4, 152.4 (Ar-C).

*2-Amino-4-phenyl-5,6-dihydrobenzo[h]quinoline-3-carbonitrile* (**8**): ^1^H-NMR (DMSO-*d*_6_) δ ppm: 2.56 (m, 2H, C-5 protons), 2.76 (m, 2H, C-6 protons), 6.45 (s, 1H, NH_2_) 7.16–7.98 (m, 9H, ArH). ^13^C-NMR (DMSO-*d*_6_) δ ppm: 25.2 (C-5), 28.5 (C-6), 117.0 (CN), 89.8, 125.5, 126.3, 126.8, 127.1, 128.4, 129.0, 138.2, 138.8, 139.2, 155.4, 160.6, 162.6 (Ar-C).

*2-Amino-4-(4-chlorophenyl)-5,6-dihydrobenzo[h]quinoline-3-carbonitrile* (**9**): ^1^H-NMR (DMSO-*d*_6_) δ ppm: 2.54 (m, 2H, C-5 protons), 2.72 (m, 2H, C-6 protons), 6.39 (s, 1H, NH_2_), 7.17–8.02 (m, 8H, ArH). ^13^C-NMR (DMSO-*d*_6_) δ ppm: 25.4 (C-5), 28.3 (C-6), 116.8 (CN), 90.0, 125.3, 126.1, 126.6, 127.1, 128.1, 129.2, 129.4, 134.3, 137.8, 138.6, 139.1, 154.4, 160.5, 162.4 (Ar-C).

#### 3.1.3. 3-(*N*,*N*-Diacetylamino)-1-(4-bromophenyl)-9,10-dihydrophenanthrene-2,4-dicarbonitriles **10**–**13**

A mixture of the appropriate 3-aminophenanthrene derivative **1**, **2**, **4** or **5** (10.00 mmol), acetic anhydride (5 mL) and conc. H_2_SO_4_ (0.2 mL) was heated in a boiling water bath for 10 min, then cooled, poured on ice-cold water, treated with 20% NaOH solution until it reaches an alkaline pH (pH~11). The resulting solid product was filtered and recrystallized from ethanol. Yields, 76%–82%; IR: (cm^−1^, KBr): 2220–2228 (CN), 1692–1705 (C=O).

*N-acetyl-N-(1-(4-bromophenyl)-2,4-dicyano-9,10-dihydrophenanthren-3-yl)acetamide* (**10**): ^1^H-NMR (DMSO-*d*_6_) δ ppm: 2.40 (s, 6H, 2CH_3_), 2.88 (m, 2H, C-9 protons), 2.84 (m, 2H, C-10 protons), 7.17–7.46 (m, 9H, Ar-H). ^13^C-NMR (DMSO-*d*_6_) δ ppm: 34.2 (C-9), 26.4 (C-10), 116.8 (CN), 97.4, 126.3, 126.5, 127.3, 127.7, 128.7, 129.2, 129.8, 136.1, 136.7, 139.1, 144.6, 151.8 (Ar-C).

*N-acetyl-N-(2,4-dicyano-1-(4-methoxyphenyl)-9,10-dihydrophenanthren-3-yl)acetamide* (**11): **^1^H-NMR (DMSO-*d*_6_) δ ppm: 2.88 (m, 2H, C-9 protons), 2.80 (m, 2H, C-10 protons), 3.72 (CH_3_O), 6.77–7.48 (m, 8H, Ar-H). ^13^C-NMR (DMSO-*d*_6_) δ ppm: 33.9 (C-9), 26.2 (C-10), 56.0 (CH_3_O), 116.7 (CN), 97.5, 114.3, 126.2, 126.4, 127.5, 128.5, 128.6, 129.6, 136.1, 139.1, 145.1, 151.9, 160.5 (Ar-C).

*N-acetyl-N-(1-(benzo[d][1,3]dioxol-5-yl)-2,4-dicyano-9,10-dihydrophenanthren-3-yl)acetamide* (**12**): ^1^H-NMR (DMSO-*d*_6_) δ ppm: 2.87 (m, 2H, C-9 protons), 2.82 (m, 2H, C-10 protons), 5.90 (CH_2_), 6.82–7.48 (m, 7H, Ar-H). ^13^C-NMR (DMSO-*d*_6_) δ ppm: 34.1 (C-9), 26.3 (C-10), 116.9 (CN), 91.2 (CH_2_), 97.4, 114.3, 115.5, 116.4, 120.5, 126.1, 127.5, 128.4, 129.3, 129.9, 136.0, 139.1, 145.4, 146.1, 148.3, 151.7 (Ar-C).

*N-acetyl-N-(2,4-dicyano-1-(thiophen-2-yl)-9,10-dihydrophenanthren-3-yl)acetamide* (**13**): ^1^H-NMR (DMSO-*d*_6_) δ ppm: 2.89 (m, 2H, C-9 protons), 2.82 (m, 2H, C-10 protons), 6.88–7.46 (m, 7H, Ar-H). ^13^C-NMR (DMSO-*d*_6_) δ ppm: 34.2 (C-9), 26.1 (C-10), 116.8 (CN), 97.1, 98.5, 122.6, 125.2, 126.2, 127.4, 127.6, 128.3, 129.2, 136.0, 139.1, 142.2, 145.4, 152.0 (Ar-C).

### 3.2. Biological Activity Evaluation

#### 3.2.1. Anti-Microbial Activity Procedure

The preliminary anti-microbial activities of compounds **1**–**13** were measured in a concentration of 50 mg/L by disc diffusion method [41,42]. The prepared compounds were tested for their antimicrobial activity against *Staphylococcus aureus* (ATCC 25923) as Gram positive bacteria, *Escherichia coli* (ATCC 25922) as Gram negative bacteria, and the antifungal activity was performed using the pathogenic yeast strains *Candida albicans* and *Aspergillus niger*. DMSO was used as a solvent and the standard drugs used were ampicillin and griseofulvin. The disc diffusion method was performed using Muller-Hinton agar (Hi-Media) medium. The inhibition zones were measured in mm at the end of an incubation period of 24 h at 37 °C for bacteria and 72 h at 24 °C for fungi. The zone of inhibition in mm is expressed in Table 2. All test data in Table 2 were of average values from triplicate run.

## 4. Conclusions

The present paper describes an efficient and simple method for the synthesis of a series of 3-amino-1-substituted-9,10-dihydrophenanthrene-2,4-dicarbonitriles **1**–**5** via one-pot multicomponent reactions (MCRs). The structures of these phenanthrene derivatives were elucidated by analytical and spectroscopic data. The conclusive proof of the phenanthrene structure was given by X-ray crystallography. The prepared compounds were screened *in vitro* for their antimicrobial and antifungal activities. The overall results suggested that compounds containing 3-(*N*,*N*-diacetylamino)- substituent exhibited relatively better antimicrobial and antifungal activities when compared with non-acetylated phenanthrene analogs, indicating a positive role of diacetyl group in the present series. Compounds **10** and **13** were however, significantly active when compared with the rest of the series. 
